# Organochloride pesticides impaired mitochondrial function in hepatocytes and aggravated disorders of fatty acid metabolism

**DOI:** 10.1038/srep46339

**Published:** 2017-04-11

**Authors:** Qian Liu, Qihan Wang, Cheng Xu, Wentao Shao, Chunlan Zhang, Hui Liu, Zhaoyan Jiang, Aihua Gu

**Affiliations:** 1 Center of Gallbladder Disease, Shanghai East Hospital, Institute of Gallstone Disease, Tongji University School of Medicine, Shanghai, 201200, China; 2 State Key Laboratory of Reproductive Medicine, Institute of Toxicology, Nanjing Medical University, Nanjing, China; 3 Key Laboratory of Modern Toxicology of Ministry of Education, School of Public Health, Nanjing Medical University, Nanjing, China; 4 Department of Surgery, Shanghai Institute of Digestive Surgery, Ruijin Hospital, Shanghai Jiao Tong University School of Medicine, Shanghai, 200025, China

## Abstract

p,p’-dichlorodiphenyldichloroethylene (p, p’-DDE) and β-hexachlorocyclohexane (β-HCH) were two predominant organochlorine pesticides (OCPs) metabolites in human body associated with disorders of fatty acid metabolism. However, the underlying mechanisms have not been fully clarified. In this study, adult male C57BL/6 mice were exposed to low dose of p, p’-DDE and β-HCH for 8 wk. OCPs accumulation in organs, hepatic fatty acid composition, tricarboxylic acid cycle (TCA) metabolites and other metabolite profiles were analyzed. Expression levels of genes involved in hepatic lipogenesis and β-oxidation were measured. Mitochondrial function was evaluated in HepG2 cells exposed to OCPs. High accumulation of p, p’-DDE and β-HCH was found in liver and damaged mitochondria was observed under electron microscopy. Expression of genes in fatty acid synthesis increased and that in mitochondrial fatty acid β-oxidation decreased in OCPs treatment groups. OCPs changed metabolite profiles in liver tissues, varied hepatic fatty acid compositions and levels of several TCA cycle metabolites. Furthermore, MitoTracker Green fluorescence, ATP levels, mitochondrial membrane potential and OCR decreased in HepG2 cells exposed to OCPs. In conclusion, chronic exposure to OCPs at doses equivalent to internal exposures in humans impaired mitochondrial function, decreased fatty acid β-oxidation and aggravated disorders of fatty acid metabolism.

Organochlorine pesticides (OCPs) are one type of persistent organic pollutants (POPs) that have caused worldwide concerns for human health. They are resistant to degradation and can accumulate at high levels in the human body^1^. Considerable quantities of these OCP compounds are detectable in our ecosphere^2^, although they were banned in the 1970s and 1980s because of their toxicity to humans.

Recent data showed that OCPs remain prevalent, worldwide, in the environment. In soil measurements, the mean concentration of total dichlorodiphenyltrichloroethane (DDT) ranged from 0.2 to 129.1 ng/g and total hexachlorocyclohexane (HCH) from 0.1 to 379.3 ng/g^3^,^4^,^5^,^6^,^7^,^8^. In air, total DDT and HCH levels ranged from 2.5 to 789.3 pg/m and 15 to 274.7 pg/m, respectively^9^,^10^,^11^. In water, 52.5 and 43.7 ng/L of total DDT and HCH, respectively, was detected^12^. Dietary intake accounted for more than 90% of the OCPs burden in the general population^13^.

Various blood levels of OCPs were documented in populations from several countries^14^,^15^,^16^. In India, reported serum levels were as high as 743 ng/mL for DDT and 627 ng/mL for HCH^16^. More surprising, OCPs were even detected in umbilical cord blood, with concentrations reaching 3.1 ng/mL for DDT and 1.1 ng/mL for HCH^17^. This suggested potential harm to fetal development during pregnancy.

Health effects of OCPs, for example, reproductive interference and immunological toxicity, were previously studied^18^. Recently, there has been more focus on the influences of OCPs on metabolism^19^,^20^,^21^. However, the underlying molecular mechanisms for how OCPs would lead to disorders in fatty acid metabolism have not yet been fully clarified.

Recently, we reported for the first time that high accumulation of both p, p’-dichlorodiphenyldichloroethylene (p, p’-DDE) and β-HCH in adipose tissues based on the population study and importantly, showed that these OCPs were associated with higher fatty acid levels in the human liver^22^. We demonstrated lipogenesis induced by both p, p’-DDE and β-HCH in hepatocytes. How these OCPs affect fatty acid degradation and other metabolic pathways, however, remains unknown. In our present study, we exposed mice to p, p’-DDE and β-HCH for 8 weeks at a dose equivalent to their internal exposure doses in humans. The underlying molecular changes contributing to disorders of hepatic fatty acid metabolism were investigated, as well as other relevant effects on hepatic metabolism.

## Results

### Accumulation of p, p’-DDE and β-HCH in the liver and alteration of hepatic fatty acid content

**Figure 1:**
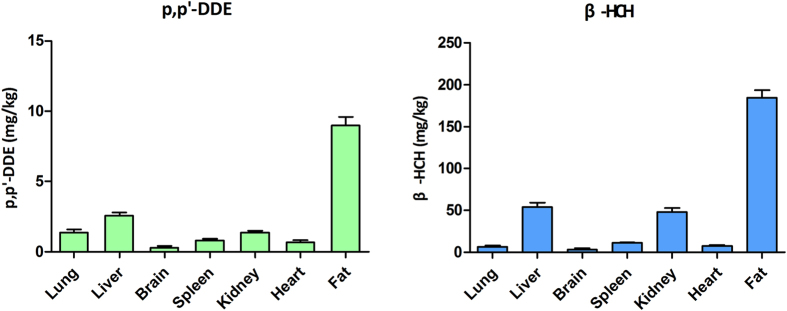
The levels of p, p’-DDE and β-HCH residues in the relevant primary organs (lung, liver, brain, spleen, kidney, heart, fat). (**A**) Exposure to p, p’-DDE, (**B**). Exposure to β-HCH, adjusted by tissue wet weight (mg/kg tissue, n = 3/group).

Eight-week exposure of mice to p, p’-DDE or β-HCH led to different levels of OCP accumulation in organs. Both compounds were abundantly detected in adipose tissues. Among parenchymal organs, liver was the primary site of p, p’-DDE and β-HCH accumulation (Fig. 1).

**Figure 2:**
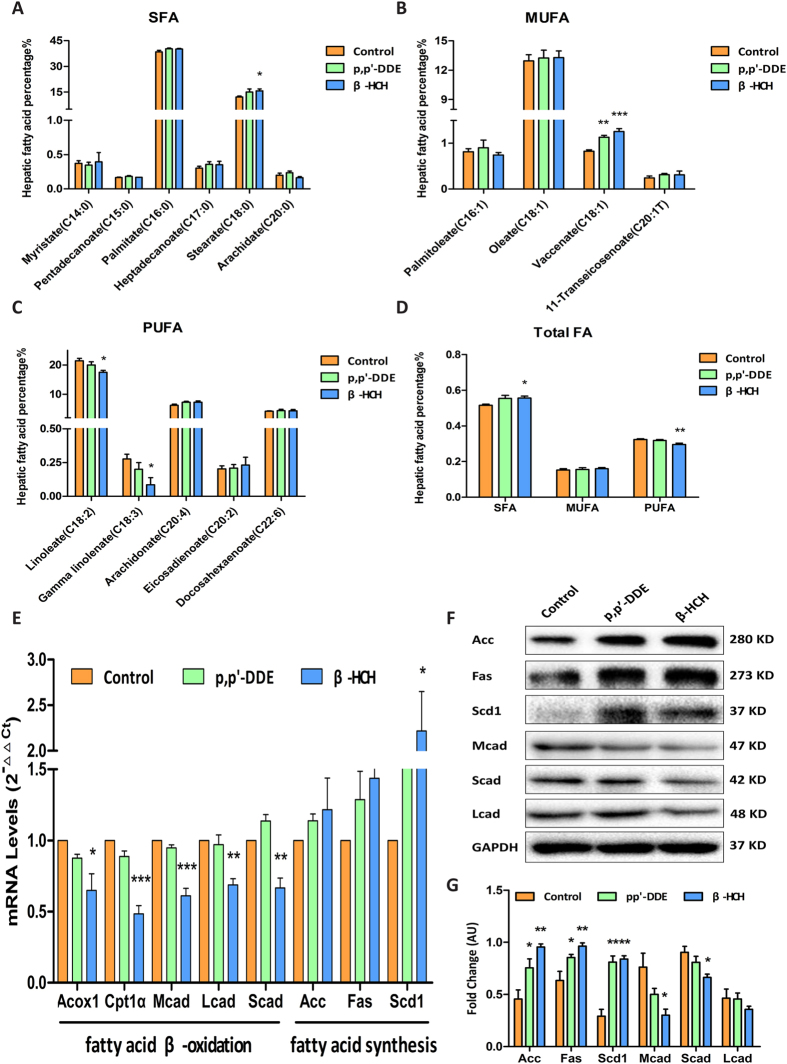
Influence on fatty acid metabolism in mouse livers exposed to p, p’-DDE and β-HCH. Quantitative analysis of individual saturated fatty acids (SFA, **A**), monounsaturated fatty acids (MUFA, **B**) and polyunsaturated fatty acids (PUFA, **C**), as well as total fatty acid content (**D**) in the liver. Expression of genes involved in hepatic fatty acid β-oxidation and synthesis (**E**), n = 5/group. Expression of Acc, Fas, Scd1, Mcad, Scad and Lcad proteins in mouse livers detected by Western blot using GAPDH as an internal control (**F** and **G**, n = 3/group). Data are expressed as means ± SEM. *P < 0.05, **P < 0.01, ***P < 0.001, compared with the control group.

β-HCH exposure led to significantly elevated levels of hepatic saturated fatty acids (SFA) and decreased polyunsaturated fatty acids (PUFA) (Fig. 2D). SFA elevation was primarily caused by stearate (C18:0) accumulation (Fig. 2A). Notably, among monounsaturated fatty acids (MUFA), vaccenate (C18:1) levels were significantly elevated (Fig. 2B). Meanwhile, decreased PUFA levels were primarily caused by decreases in linoleate (C18:2) and γ-linolenate (C18:3) (Fig. 2C).

There were no differences in serum liver enzymes levels (Supplement Table 2) and increment of body weight among groups (Fig. S1).

### Pathological changes in liver tissues

**Figure 3:**
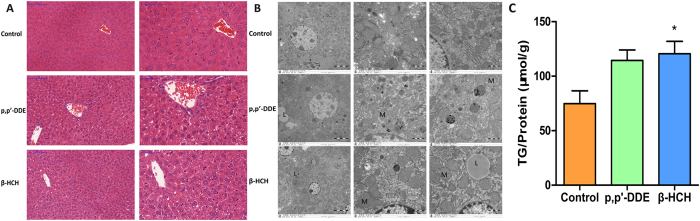
(**A**) Representative haematoxylin and eosin staining revealed gradual enhancement of hepatocellular fatty degeneration with looser cell gaps and arrangements (scale bars: 100 μm, 50 μm) in liver tissue from mice exposed to p, p’-DDE or β-HCH. (**B**) Representative electron micrographs of liver from mice exposed to p, p’-DDE or β-HCH (6000×, 25000×). Mitochondria (M) were mildly enlarged, matrices were electron-lucent and cristae were damaged. Lipid droplets (L) formed and loose organelle gaps were observed in OCP treated groups. (**C**) TG content in livers of mice exposed to p, p’-DDE or β-HCH (n = 5/group). The data are presented as the mean ± SEM. *P < 0.05 compared with the control group.

Haemotoxylin eosin (HE) staining showed cytoplasmic vacuolation of peri-portal and centri-lobular hepatocytes in mouse liver after exposed to p, p’-DDE or β-HCH (Fig. 3A). Scattered clusters of mitochondria were mildly to markedly enlarged and often irregularly shaped in the majority of hepatocytes after chronic p, p’-DDE or β-HCH exposure. The mitochondria cristae had been markedly damaged, with relatively abnormal mitochondrial architecture. Moreover, a number of lipid droplets were observed in OCPs exposure groups (Fig. 3B). Hepatic TG level increased after β-HCH exposure (Fig. 3C) as well as a trend after p, p’-DDE exposure.

### Decreased expression of genes involved in mitochondrial fatty acid β-oxidation

We then measured mRNA expression of key enzymes for mitochondrial fatty acid β-oxidation and fatty acid synthesis. As shown in Fig. 2E, mRNA expression of carnitine palmitoyl transferase 1α (Cpt1α), short chain acyl-CoA dehydrogenase (Scad), medium chain acyl-CoA dehydrogenase (Mcad) and long chain acyl-CoA dehydrogenase (Lcad), enzymes responsible for fatty acid β-oxidation, were significantly lower in the β-HCH group (Fig. 2E). The differences were further confirmed at the protein level (Fig. 2F and G). These findings suggested impairment of mitochondrial fatty acid β-oxidation, consistent with the pathological defects seen in mitochondria. Furthermore, mRNA levels of fatty acid synthase (Fas), acetyl-CoA carboxylase (Acc) and stearoyl CoA desaturase 1 (Scd1) were higher in both p, p’-DDE and β-HCH groups (Fig. 2E), suggesting enhanced hepatic lipogenesis in mice exposed to OCPs, consistent with our previous observations in human liver^22^.

### Changes in hepatic tricarboxylic acid (TCA) cycle metabolites

**Figure 4:**
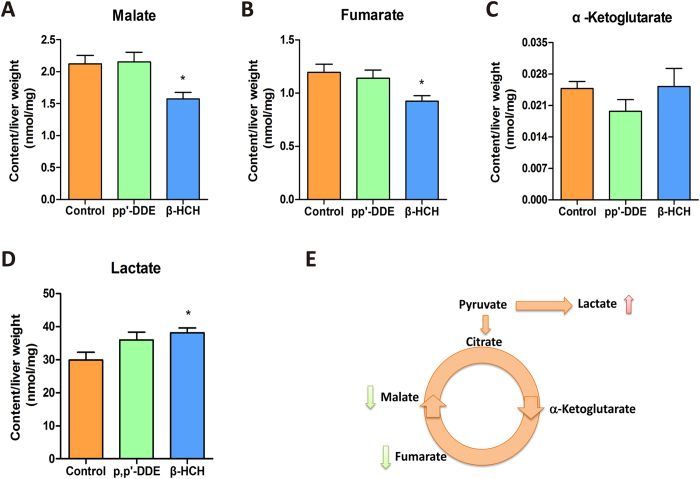
TCA cycle metabolites in livers of mice exposed to p, p’-DDE or β-HCH. Contents of malate (**A**), fumarate (**B**), α-ketoglutarate (**C**) and lactate (**D**), n = 5/group. (**E**) A schematic diagram showing the TCA cycle. Data are expressed as means ± SEM. *P < 0.05 compared with the control group.

The metabolites involved in the mitochondrial TCA cycle in liver were measured by GC/MS. Malate and fumarate levels significantly decreased in mice exposed to β-HCH (Fig. 4C and D). In contrast, more pyruvate was converted into lactate (Fig. 4B). These results suggested the inefficiency in hepatic mitochondrial TCA cycle in these mice.

### Changes in liver metabolomics profiles

**Figure 5:**
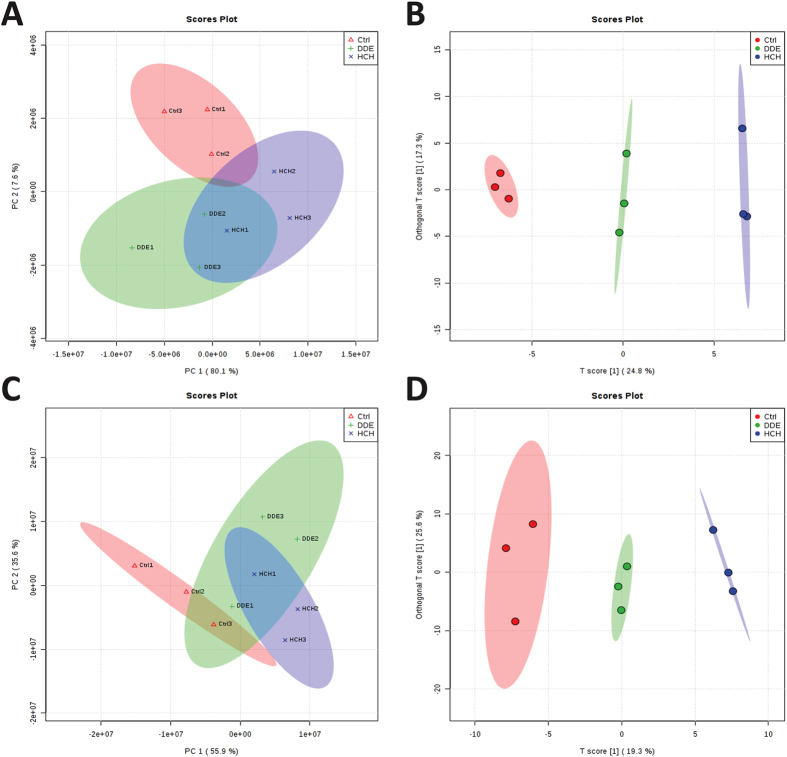
Metabolite profiles in liver tissues from control, p, p’-DDE and β-HCH groups, obtained by LC/MS analysis in positive (**A** and **B**) and negative (**C** and **D**) ion modes. (**A** and **C**) PCA score plots; (**B** and **D**) OPLS-DA score plots.

Changes in hepatic metabolomics after p, p’-DDE or β-HCH exposure were further analyzed by LC/MS. Principal component analysis (PCA) and orthogonal projections to latent structures discriminant analysis (OPLS-DA) score plots of both positive and negative mode data revealed distinct metabolite profiles in liver tissues from p, p’-DDE, β-HCH and control groups (Fig. 5). The fold-changes of identified metabolites after p, p’-DDE and β-HCH exposures are presented in Supplement Table 3. It was very interesting that trends for changes in the majority of metabolites were similar for the two OCP compounds. The identity of these affected metabolites implied perturbations in phospholipid, fatty acid and amino acid metabolism.

### Impairment of mitochondrial function and fatty acid β-oxidation by p, p’-DDE and β-HCH in HepG2 cells

**Figure 6:**
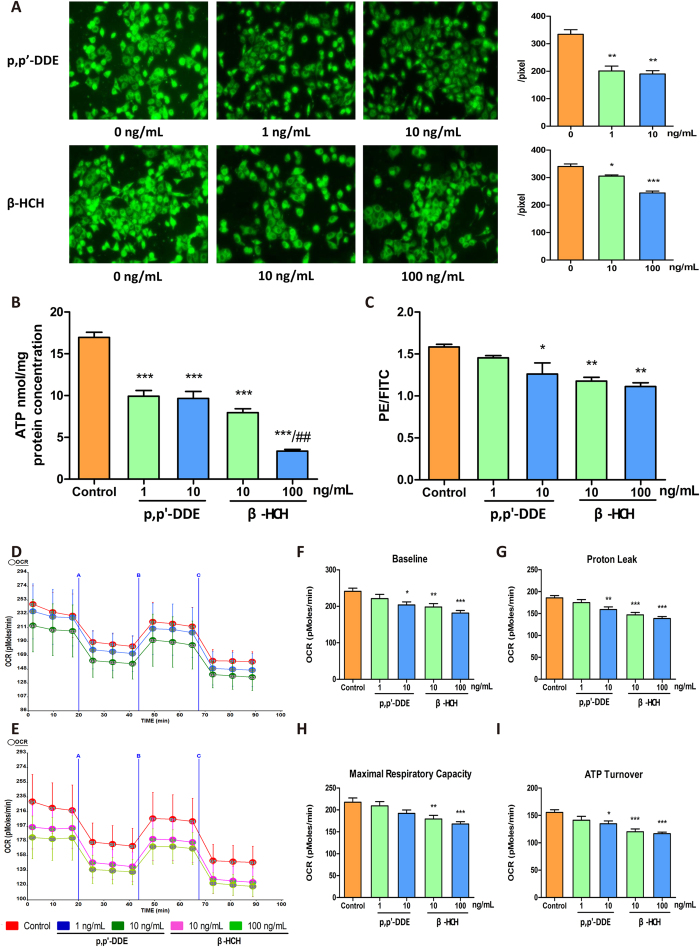
Effects on mitochondrial function in HepG2 cells treated with p, p’-DDE or β-HCH. (**A**) Fluorescence microscopic images showing amount, location and fluorescence intensity of mitochondria and quantitative levels of mitochondrial fluorescence intensity per cell, based on MitoTracker Green staining. (**B**) ATP levels in HepG2 cells. (**C**) Detection of mitochondrial membrane potential by JC-1 staining and flow cytometry. The Y-axis shows the ratio of red to green fluorescence. (**D** and **E**) Effects on cellular oxygen consumption rate (OCR). Quantitative histogram of OCR results for baseline (**F**), proton leak (**G**), maximal respiratory capacity (**H**) and ATP turnover (**I**). *P < 0.05, **P < 0.01, ***P < 0.001 compared with the control, ^##^*P* < 0.01 compared with 10 ng/mL β-HCH. Each data point is the mean ± SEM from three separate experiments.

Fluorescence intensity of MitoTracker Green which labelled mitochondria decreased (Fig. 6A), as well as ATP levels, in HepG2 cells treated with p, p’-DDE or β-HCH (Fig. 6B). This was potentially caused by both decreased mitochondria number and TCA cycle deficiency, leading to an overall decrease in ATP production. Flow cytometry results showed that the ratios of aggregate/monomer (red/green JC-1 fluorescence) in the treated groups were significantly lower than in the control (Fig. 6C) which indicated that p, p’-DDE and β-HCH decreased mitochondrial membrane potential.

The oxygen consumption rate (OCR) in HepG2 cells was decreased in a dose-dependent manner after p, p’-DDE (Fig. 6D) or β-HCH (Fig. 6E) treatments. Compared with control cells, basal respiration rates were significantly decreased, by 15.5% with 10 ng/mL p, p’-DDE and by 17.8 and 24.8% with 10 and 100 ng/mL β-HCH treatments, respectively (Fig. 6F). Furthermore, proton leak, maximal respiratory capacity and ATP turnover were significantly declined in p, p’-DDE (10 ng/mL) and β-HCH (10 and 100 ng/mL) exposed groups, compared with control group (Fig. 6G–I). These results indicated that there was mitochondrial dysfunction after OCP treatments although there was no notable cytotoxicity at that doses in HepG2 cells (Fig. S2).

**Figure 7:**
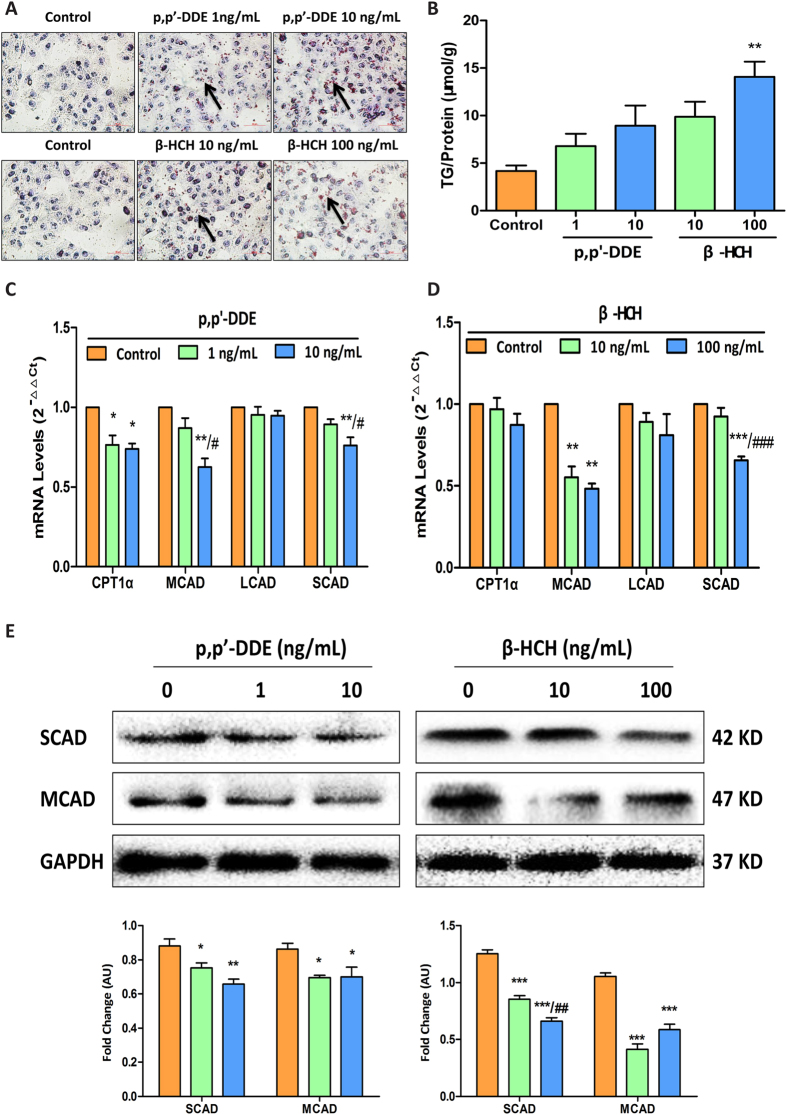
Effects on lipid metabolism in HepG2 cells treated with p, p’-DDE or β-HCH. (**A**) Oil Red O staining of HepG2 cells. Accelerated lipid accumulation in HepG2 cells treated with p, p’-DDE or β-HCH. Red dot (arrowhead) shows a lipid droplet and pale blue circle shows the cell nucleus. Cells were treated with p, p’-DDE or β-HCH for 24 h. Cells were observed under a magnification of 40×, with scale bar indicating 50 μm. (**B**) Cellular TG content after 24-hour incubation with p, p’-DDE or β-HCH. C and D: Changes in mRNA expression of genes involved in hepatic fatty acid β-oxidation in HepG2 cells incubated with p, p’-DDE (**C**) or β-HCH (**D**). (**E**) Protein levels of SCAD and MCAD in HepG2 cells incubated with p, p’-DDE and β-HCH detected by Western blot using GAPDH as an internal control. *P < 0.05, **P < 0.01, ***P < 0.001 compared with the control, ^#^*P* < 0.05 compared with 1 ng/mL p, p’-DDE, ^##^*P* < 0.01 compared with 10 ng/mL β-HCH, ^###^*P* < 0.001 compared with 10 ng/mL β-HCH. Each data point represents the mean ± SEM from three separate experiments.

Oil Red O staining in cells after incubation with either OCP indicated cellular lipid accumulation (Fig. 7A), which was further confirmed by increased TG content in cells (Fig. 7B). The expression of CPT1α, SCAD and MCAD, enzymes responsible for fatty acid β-oxidation, were significantly lower in cells treated with p, p’-DDE or β-HCH (Fig. 7C–E).

## Discussion

**Figure 8:**
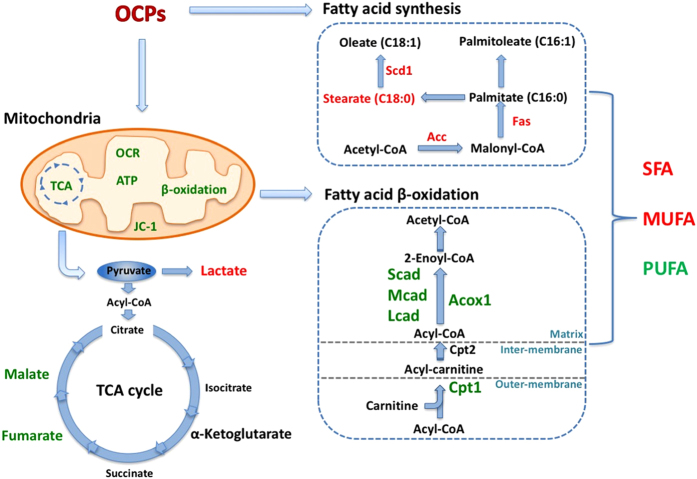
Schematic summary of potential biological hazards of OCPs (p, p’-DDE and β-HCH) and mechanism of their effects on hepatic accumulation of fatty acids, through damage of mitochondrial function in hepatocytes, decreased fatty acid β-oxidation and increased fatty acid synthesis.

We recently found that p,p’-DDE and β-HCH were two predominant OCPs metabolites among all different OCPs detected in human body and they were associated with disorders of lipid metabolism^22^. In this study, we for the first time showed that these two representative OCPs could lead to changes in metabolite profile in liver (Fig. 5), impair hepatic mitochondria function (Fig. 6) and disrupt fatty acid β-oxidation and TCA cycle (schematically shown in Fig. 8). Deficient fatty acid β-oxidation and enhanced lipogenesis in concert led to increased hepatic SFA and decreased PUFA levels (Fig. 2).

Our previous population study^22^ and others^23^ found that disorders of fatty acid metabolism caused by OCPs were partially due to induction of lipogenesis through activation of SREBP1c-regulation^24^. However, no report ever examined the influence of OCPs on fatty acid degradation. The findings in this study provided another mechanism that impaired mitochondrial fatty acid β-oxidation by OCPs played a role in the disorders in hepatic fatty acid metabolism. Both defects could result in increased SFA and decreased PUFA levels in the liver, the metabolic changes similar as observed in rats^23^. The major risk of fatty acid accumulation is its potential to provide excessive substrates for triglyceride synthesis and then associated with steatosis^25^. Moreover, cholesteryl esters derived from SFA were reported to be atherogenic^26^.

Our findings that OCPs decreased mitochondria number and ATP levels were consistent with previous observations in rats^27^. In addition, we provided new evidences for mitochondria dysfunction, including decreased membrane potential, defects in OCR and decreased ATP production in OCP exposure group. Meanwhile, the expression of key enzymes in mitochondrial fatty acid β-oxidation decreased and TCA cycle was defected. Modulation of the TCA cycle by β-HCH has been reported during hepato-carcinogenesis^28^. We observed similar effects on hepG2 cells by p, p’-DDE and β-HCH. This implied impairment of mitochondrial function as an important and early event accounting for the hepatic metabolic disorders caused by OCPs, prior to detectable biochemical abnormalities.

In previous studies which investigating the toxicological effect of OCPs in rodents, the doses of p, p’-DDE and β-HCH used ranged from 2–50 mg/kg^29^,^30^,^31^ and 100–500 mg/kg^32^,^33^, respectively. To mimic internal exposures in the human body, we selected low doses in this study that were close to the low level of the range detected in humans and environment^12^,^34^,^35^. Such doses did not cause abnormalities in serum liver enzymes, but subcellular pathological changes as well as changes in metabolites profiles in liver tissues already occurred. To those non-occupationally populations who are usually exposed to low but persistent environmental OCPs, our findings might give more information about the low-dose effects on metabolism in the body. Adipose tissues tended to have highest accumulation of OCPs in the body similar as we observed in this study and the levels increased with exposure time^36^. In human, the OCPs levels were only measured in serum or adipose tissues due to the availability of samples. Though the values in human adipose tissues varied between studies, they could reach up to 19 mg/kg^37^ for p,p’-DDE and 33.9 mg/kg^38^ for β-HCH in non-occupational populations. Furthermore, levels of OCPs were fold higher in population living in the area of potential source of environmental contaminations^39^. Therefore, the OCPs accumulated in adipose tissue might serve as a source of contaminants reaching liver through constant releasing into circulation.

In conclusion, the present study updated our understanding of p’-DDE and β-HCH and their contributions to disorders of hepatic lipid metabolism. They showed a tendency to accumulate in the liver, impairing mitochondrial function and leading to changes of hepatic metabolites profiles. In addition to inducing lipogenesis in hepatocytes, p, p’-DDE and β-HCH can disrupt mitochondrial fatty acid β-oxidation, in turn, aggravating disorders of fatty acid metabolism in the liver (Fig. 8). Since OCPs could lead to changes in multiple physiological processes involved in lipid metabolic disorder, our results suggested a need for awareness of environmental OCP exposure and its metabolic effects in various populations.

## Methods

### Chemicals

p, p’-DDE was from Tokyo Chemical Industry (Shanghai, China, B0133-1 g, purity >99.0%, CAS No. 72-55-9). β-HCH was from Aladdin (Shanghai, China, H114177-100 mg, CAS No. 319-85-7). Both were of research grade.

### Animal experiment procedures

Male adult C57BL/6 mice (Shanghai SLAC Laboratory Animal Co., Ltd. Shanghai, China) were fed a standard chow diet. They were treated with p, p’-DDE (1 mg/kg/day, DDE group), β-HCH (10 mg/kg/day, HCH group) or vehicle (control group), each administered by oral gavage once per day for 8 wk (n = 8/group). On the day of sacrifice, blood samples were collected from all mice by the retro-orbital venous sinus and key organs (lung, liver, brain, spleen, kidney, heart, fat) were harvested. The experiment protocol was approved by the local Ethical Committee of Nanjing Medical University. In addition, all experiments were performed in accordance with relevant guidelines and regulations.

### Determination of clinical biochemical indicators in serum and accumulation of p, p’-DDE and β-HCH levels in organs

Biochemical indicators of liver and kidney function, serum lipids and glucose were measured with an automatic biochemical analyser. Accumulation of p, p’-DDE and β-HCH in various organs was determined using an Agilent 7890 A gas chromatography mass spectrometry (GC-MS) (Agilent Technologies, Santa Clara, CA, USA) as previously described^22^.

### Haematoxylin and eosin (HE) staining, electron microscopy and Oil Red O staining

Paraffin-embedded livers were cut into section of 5 μm thickness and stained with haematoxylin and eosin (HE). Liver tissue samples were also examined on the JEOL-1010 transmission electron microscope. For Oil Red O staining, HepG2 cells treated with p, p’-DDE or β-HCH were fixed in a 10% formalin solution and stained with Oil Red O (Nanjing Senbeijia Biological Technology Co., Ltd. Nanjing, China).

### Assay of triglycerides content

Triglyceride (TG) content was measured by a colorimetric assay (Applygen Technologies Inc., Beijing, China) using liver homogenate or HepG2 cells after lipid extraction by chloroform and methanol (1:3, v/v). All samples were determined in duplicate and TG values were expressed as μmol of TG/g of protein.

### Measurement of hepatic metabolite profiles and levels of fatty acids and tricarboxylic acid (TCA) cycle metabolites in liver

Hepatic metabolite profiles were analysed using an Agilent 1290 Infinity Liquid Chromatography System (Agilent Technologies) equipped with a 2.1 × 100 mm C18 reverse-phase column with 1.8-μm particle size (Waters Corp., Milford, MA, USA) as described previously^40^. Mass spectrometry was performed on an Agilent 6530 Accurate-Mass QTOF/MS (Agilent Technologies) equipped with an electrospray ionisation source. Data for each ionisation technique were acquired in positive and negative ion modes. LC data were acquired and processed using Mass Hunter Qualitative Analysis Software (version B.03.01; Agilent Technologies). The MS analysis system was used to identify metabolites corresponding to those in the METLIN database (http://metlin.scripps.edu). SIMCA-P+ 11.0 software (Umetrics AB, Umea, Sweden) and online tool MetaboAnalyst 3.0 (http://www.metaboanalyst.ca/MetaboAnalyst) were used for PCA, partial least squares discriminant analysis (PLS-DA) and OPLS-DA analyses. A t-test was used to identify those candidate metabolites obtained from PLS-DA modelling that were statistically different from those in the control group.

Fatty acids in liver tissue were measured by gas chromatography as previously described^41^,^42^. TCA cycle metabolites in liver tissue were assayed with a Shimadu QP-2010 ultra GC/MS^43^.

### Determining mRNA expression of genes involved in lipid metabolism by quantitative real-time PCR

Total RNA was isolated from liver tissues or cells with TRIzol (Invitrogen, Carlsbad, CA, USA). cDNA was synthesised with PrimeScript™ RT Master Mix (Takara, Dalian, China). Quantitative real-time PCR with SYBR Green was performed with an ABI 7900 HT fast real-time system (Applied Biosystems, Foster City, CA, USA). Relative mRNA expression was calculated by the 2^−ΔΔCt^ method using GAPDH as the internal control. The primer sequences are listed in Supplement Table 1.

### Detection of proteins involved in fatty acid metabolism by Western blot

Total proteins of cell lysate or liver homogenates were separated on SDS-PAGE, then transferred to polyvinylidene fluoride membranes (Millipore, Billerica, MA, USA). The antibodies used in western blot assay were anti-Acetyl-CoA Carboxylase (ACC); anti-Fatty Acid Synthase (FAS), and anti-SCD1 antibodies (Cell Signaling Technology, 1:1000); anti-Mcad, anti-LCAD and anti- SCAD antibodies (Abcam, 1:1000). The immune complexes were detected by enhanced chemiluminescence (Millipore, Billerica, MA, USA). Anti-GAPDH (Beyotime, 1:1000) was as an internal control. The band was quantified using Image Lab software (BioRad laboratories, Hercules, CA, USA). Each experiment was performed at least twice.

### Mitochondrial function in hepatocytes

HepG2 cells were cultured in DMEM supplemented with penicillin/streptomycin and 10% fetal bovine serum (FBS). When cells reached 50% confluence, p, p’-DDE (0, 1, 10 ng/mL) or β-HCH (0, 10, 100 ng/mL) were added. Cell viability was determined with a cell counting Kit-8 (CCK-8) assay (Vazyme Biotech Co.,Ltd. Nanjing, China).

After HepG2 cells were incubated with p, p’-DDE and β-HCH for 24 h, MitoTracker Green solution (final concentration: 20 nM, Beyotime, Haimen, China) was added and cells incubated at 37 °C for 45 min. Mitochondrial green fluorescence intensity was observed with a fluorescence microscope. ATP levels in cells were determined with a luciferase-luciferin ATP assay kit (Beyotime, Haimen, China) and the ATP contents normalised to protein concentrations. The cationic dye JC-1 was used to detect the mitochondrial membrane potential in HepG2 cells incubated with p, p’-DDE or β-HCH, according to the protocol provided with the mitochondrial membrane potential assay kit (Beyotime, Haimen, China). Oxygen consumption rates (OCR) were measured with a Seahorse XF96 Extracellular Flux analyser (Seahorse Bioscience, North Billerica, MA, USA) to assess mitochondrial dysfunction. In brief, after baseline measurements of OCR, OCR was measured after sequentially adding to each well oligomycin, FCCP and antimycin A/rotenone. OCR was automatically recorded by the XF96 software.

### Data analysis

All experiments were performed in triplicate and repeated at least twice. All values are presented as means ± SEM of the indicated number of independent experiments.

Statistical significances of multiple treatments were determined by one-way ANOVA and Bonferoni or Dunnett’s multiple comparison test with SPSS 20.0. A *P* value < 0.05 was designated as statistically significant.

## Additional Information

**How to cite this article:** Liu, Q. *et al*. Organochloride pesticides impaired mitochondrial function in hepatocytes and aggravated disorders of fatty acid metabolism. *Sci. Rep.* **7**, 46339; doi: 10.1038/srep46339 (2017).

**Publisher's note:** Springer Nature remains neutral with regard to jurisdictional claims in published maps and institutional affiliations.

## Supplementary Material

Supplementary Tables and Figures

## References

[b1] Maisano M. . PCB and OCP accumulation and evidence of hepatic alteration in the Atlantic bluefin tuna, T. thynnus, from the Mediterranean Sea. Mar Environ Resdoi: 10.1016/j.marenvres.2016.03.003 (2016). 27012897

[b2] Minh T. B. . Isomer-specific accumulation and toxic assessment of polychlorinated biphenyls, including coplanar congeners, in cetaceans from the North Pacific and Asian coastal waters. Archives of environmental contamination and toxicology 39, 398–410 (2000). 10948292 10.1007/s002440010121

[b3] Sun J. . Contamination of phthalate esters, organochlorine pesticides and polybrominated diphenyl ethers in agricultural soils from the Yangtze River Delta of China. Sci Total Environ 544, 670–676, doi: 10.1016/j.scitotenv.2015.12.012 (2016). 26674696

[b4] Wang B. . Levels and patterns of organochlorine pesticides in agricultural soils in an area of extensive historical cotton cultivation in Henan province, China. Environ Sci Pollut Res Int 23, 6680–6689, doi: 10.1007/s11356-015-5864-x (2016). 26645233

[b5] Liu Q., Tian S., Jia R. & Liu X. Pollution characteristics and ecological risk assessment of HCHs and DDTs in estuary wetland sediments from the Bohai Bay, North China. Environ Sci Pollut Res Intdoi: 10.1007/s11356-015-5882-8 (2015). 26645229

[b6] Yu Y. . Occurrence and possible sources of organochlorine pesticides (OCPs) and polychlorinated biphenyls (PCBs) along the Chao River, China. Chemosphere 114, 136–143, doi: 10.1016/j.chemosphere.2014.03.095 (2014). 25113194

[b7] Huang T. . Assessing spatial distribution, sources, and human health risk of organochlorine pesticide residues in the soils of arid and semiarid areas of northwest China. Environ Sci Pollut Res Int 21, 6124–6135, doi: 10.1007/s11356-014-2505-8 (2014). 24474559

[b8] Wong F., Robson M., Diamond M. L., Harrad S. & Truong J. Concentrations and chiral signatures of POPs in soils and sediments: a comparative urban versus rural study in Canada and UK. Chemosphere 74, 404–411, doi: 10.1016/j.chemosphere.2008.09.051 (2009). 19022474

[b9] Bajwa A. . Organochlorine pesticides (OCPs) in the Indus River catchment area, Pakistan: Status, soil-air exchange and black carbon mediated distribution. Chemosphere 152, 292–300, doi: 10.1016/j.chemosphere.2016.01.024 (2016). 26978705

[b10] Munoz-Arnanz J., Roscales J. L., Ros M., Vicente A. & Jimenez B. Towards the implementation of the Stockholm Convention in Spain: Five-year monitoring (2008–2013) of POPs in air based on passive sampling. Environ Pollutdoi: 10.1016/j.envpol.2016.01.052 (2016). 26905212

[b11] Takazawa Y., Takasuga T., Doi K., Saito M. & Shibata Y. Recent decline of DDTs among several organochlorine pesticides in background air in East Asia. Environ Pollutdoi: 10.1016/j.envpol.2016.02.019 (2016). 26896161

[b12] Wu C., Luo Y., Gui T. & Huang Y. Concentrations and potential health hazards of organochlorine pesticides in (shallow) groundwater of Taihu Lake region, China. Sci Total Environ 470–471, 1047–1055, doi: 10.1016/j.scitotenv.2013.10.056 (2014). 24239826

[b13] Appenzeller B. M. & Tsatsakis A. M. Hair analysis for biomonitoring of environmental and occupational exposure to organic pollutants: state of the art, critical review and future needs. Toxicol Lett 210, 119–140, doi: 10.1016/j.toxlet.2011.10.021 (2012). 22079616

[b14] Schettgen T., Alt A., Esser A. & Kraus T. Current data on the background burden to the persistent organochlorine pollutants HCB, p,p’-DDE as well as PCB 138, PCB 153 and PCB 180 in plasma of the general population in Germany. Int J Hyg Environ Health 218, 380–385, doi: 10.1016/j.ijheh.2015.02.006 (2015). 25777936

[b15] Elbashir A. B., Abdelbagi A. O., Hammad A. M., Elzorgani G. A. & Laing M. D. Levels of organochlorine pesticides in the blood of people living in areas of intensive pesticide use in Sudan. Environmental monitoring and assessment 187, 68, doi: 10.1007/s10661-015-4269-0 (2015). 25647792

[b16] Mishra K., Sharma R. C. & Kumar S. Organochlorine pollutants in human blood and their relation with age, gender and habitat from North-east India. Chemosphere 85, 454–464, doi: 10.1016/j.chemosphere.2011.07.074 (2011). 21925698

[b17] Cao L. L. . Relationship between serum concentrations of polychlorinated biphenyls and organochlorine pesticides and dietary habits of pregnant women in Shanghai. Sci Total Environ 409, 2997–3002, doi: 10.1016/j.scitotenv.2011.04.040 (2011). 21665017

[b18] Mrema E. J. . Persistent organochlorinated pesticides and mechanisms of their toxicity. Toxicology 307, 74–88, doi: 10.1016/j.tox.2012.11.015 (2013). 23219589

[b19] Dirinck E. . Obesity and persistent organic pollutants: possible obesogenic effect of organochlorine pesticides and polychlorinated biphenyls. Obesity (Silver Spring) 19, 709–714, doi: 10.1038/oby.2010.133 (2011). 20559302

[b20] Lee D. H., Lee I. K., Jin S. H., Steffes M. & Jacobs D. R. Jr. Association between serum concentrations of persistent organic pollutants and insulin resistance among nondiabetic adults: results from the National Health and Nutrition Examination Survey 1999–2002. Diabetes Care 30, 622–628, doi: 10.2337/dc06-2190 (2007). 17327331

[b21] Rantakokko P. . Persistent organic pollutants and non-alcoholic fatty liver disease in morbidly obese patients: a cohort study. Environ Health 14, 79, doi: 10.1186/s12940-015-0066-z (2015). 26420011 PMC4588245

[b22] Ji G. . Organochloride pesticides induced hepatic ABCG5/G8 expression and lipogenesis in Chinese patients with gallstone disease. Oncotargetdoi: 10.18632/oncotarget.9399 (2016). PMC5085112 27203212

[b23] Rodriguez-Alcala L. M. . Endocrine Disruptor DDE Associated with a High-Fat Diet Enhances the Impairment of Liver Fatty Acid Composition in Rats. J Agric Food Chem 63, 9341–9348, doi: 10.1021/acs.jafc.5b03274 (2015). 26449595

[b24] Ruzzin J. . Persistent organic pollutant exposure leads to insulin resistance syndrome. Environ Health Perspect 118, 465–471, doi: 10.1289/ehp.0901321 (2010). 20064776 PMC2854721

[b25] Marchesini G., Petta S. & Dalle Grave R. Diet, weight loss, and liver health in nonalcoholic fatty liver disease: Pathophysiology, evidence, and practice. Hepatology 63, 2032–2043, doi: 10.1002/hep.28392 (2016). 26663351

[b26] Ma J., Folsom A. R., Lewis L. & Eckfeldt J. H. Relation of plasma phospholipid and cholesterol ester fatty acid composition to carotid artery intima-media thickness: the Atherosclerosis Risk in Communities (ARIC) Study. Am J Clin Nutr 65, 551–559 (1997). 9022543 10.1093/ajcn/65.2.551

[b27] Mota P. C. . Differential effects of p,p’-DDE on testis and liver mitochondria: implications for reproductive toxicology. Reprod Toxicol 31, 80–85, doi: 10.1016/j.reprotox.2010.09.010 (2011). 20951795

[b28] Bhatt D. K. & Bano M. Modulation of tricarboxylic acid cycle dehydrogenases during hepatocarcinogenesis induced by hexachlorocyclohexane in mice. Exp Toxicol Pathol 61, 325–332, doi: 10.1016/j.etp.2008.09.004 (2009). 18951770

[b29] Cetkovic-Cvrlje M., Olson M., Schindler B. & Gong H. K. Exposure to DDT metabolite p,p’-DDE increases autoimmune type 1 diabetes incidence in NOD mouse model. J Immunotoxicol 13, 108–118, doi: 10.3109/1547691X.2015.1017060 (2016). 25721050

[b30] Howell G. E. 3rd, Mulligan C., Meek E. & Chambers J. E. Effect of chronic p,p’-dichlorodiphenyldichloroethylene (DDE) exposure on high fat diet-induced alterations in glucose and lipid metabolism in male C57BL/6H mice. Toxicology 328, 112–122, doi: 10.1016/j.tox.2014.12.017 (2015). 25541407 PMC6490679

[b31] Ter Veld M. G. . Food-associated estrogenic compounds induce estrogen receptor-mediated luciferase gene expression in transgenic male mice. Chem Biol Interact 174, 126–133, doi: 10.1016/j.cbi.2008.03.019 (2008). 18501883

[b32] Prabhakaran S. & Devi K. S. Impact of protein deficiency and exposure to hexachlorocyclohexane or malathion on lipid metabolism in pregnant rats. Indian J Biochem Biophys 30, 234–238 (1993). 7506231

[b33] Cornacoff J. B. . Evaluation of the immunotoxicity of beta-hexachlorocyclohexane (beta-HCH). Fundam Appl Toxicol 11, 293–299 (1988). 2464515 10.1016/0272-0590(88)90154-6

[b34] Chu S., Covaci A. & Schepens P. Levels and chiral signatures of persistent organochlorine pollutants in human tissues from Belgium. Environ Res 93, 167–176 (2003). 12963401 10.1016/s0013-9351(03)00016-1

[b35] Park M. J. . Distribution of organochlorines and PCB congeners in Korean human tissues. Arch Pharm Res 28, 829–838 (2005). 16114499 10.1007/BF02977350

[b36] Howell G. E. 3rd . Exposure to p,p’-dichlorodiphenyldichloroethylene (DDE) induces fasting hyperglycemia without insulin resistance in male C57BL/6H mice. Toxicology 320, 6–14, doi: 10.1016/j.tox.2014.02.004 (2014). 24582731 PMC4098932

[b37] Nakata H. . Organochlorine pesticides and polychlorinated biphenyl residues in foodstuffs and human tissues from china: status of contamination, historical trend, and human dietary exposure. Archives of environmental contamination and toxicology 43, 473–480, doi: 10.1007/s00244-002-1254-8 (2002). 12399919

[b38] Aulakh R. S. . Occurrence of DDT and HCH insecticide residues in human biopsy adipose tissues in Punjab, India. Bulletin of environmental contamination and toxicology 78, 330–334, doi: 10.1007/s00128-007-9187-6 (2007). 17618386

[b39] Chovancova J. . Polychlorinated biphenyls and selected organochlorine pesticides in serum of Slovak population from industrial and non-industrial areas. Environmental monitoring and assessment 186, 7643–7653, doi: 10.1007/s10661-014-3956-6 (2014). 25098899

[b40] Chen Y. . Biomarker identification and pathway analysis by serum metabolomics of lung cancer. Biomed Res Int 2015, 183624, doi: 10.1155/2015/183624 (2015). 25961003 PMC4415745

[b41] Yao X. . Regulation of fatty acid composition and lipid storage by thyroid hormone in mouse liver. Cell Biosci 4, 38, doi: 10.1186/2045-3701-4-38 (2014). 25105012 PMC4124172

[b42] Zong G. . Associations of erythrocyte palmitoleic acid with adipokines, inflammatory markers, and the metabolic syndrome in middle-aged and older Chinese. Am J Clin Nutr 96, 970–976, doi: 10.3945/ajcn.112.040204 (2012). 23015321 PMC3471208

[b43] Scott D. A. . Comparative metabolic flux profiling of melanoma cell lines: beyond the Warburg effect. J Biol Chem 286, 42626–42634, doi: 10.1074/jbc.M111.282046 (2011). 21998308 PMC3234981

