# Supplementary material for: Organochloride pesticides impaired mitochondrial function in hepatocytes and aggravated disorders of fatty acid metabolism
Source: Sci Rep. 2017 Apr 11;7:46339. doi: 10.1038/srep46339 (PMC5387717; doi:10.1038/srep46339)
Supplement: Supplementary Tables and Figures [file srep46339-s1.pdf]

Supplementary Materials

Organochloride pesticides impaired mitochondrial function in hepatocytes and aggravated disorders of fatty acids metabolism

Qian Liu[1,2,3*], Qihan Wang[4*], Cheng Xu[2,3], Wentao Shao[2,3], Chunlan Zhang[2,3], Hui Liu[2,3], Zhaoyan Jiang[1,4], Aihua Gu[2,3]

[1] Center of Gallbladder Disease, Shanghai East Hospital, Institution of Gallstone Disease, Tongji University School of Medicine, Shanghai, China, 201200
[2] State Key Laboratory of Reproductive Medicine, Institute of Toxicology, Nanjing Medical University, Nanjing, China
[3] Key Laboratory of Modern Toxicology of Ministry of Education, School of Public Health, Nanjing Medical University, Nanjing, China
[4] Department of Surgery, Shanghai Institute of Digestive Surgery, Ruijin Hospital, Shanghai Jiao Tong University School of Medicine, Shanghai, China, 200025

* These authors contributed equally to this study and all should be considered as first authors.

§ **Corresponding authors:**
Aihua Gu: aihuagu@njmu.edu.cn
Zhao-Yan Jiang: zhaoyanjiang@gmail.com

Supplement Table 1. Sequences of primers for RT-PCR.

Supplement Table 2. Effect of p,p'-DDE and β-HCH on serum biochemical

Supplement Table 3. Metabolomic analysis of liver tissues from mouse exposed to OCPs

Supplementary Figure Legends

Figure S1 Changes in body weight among treatment groups (n=8/group).

Figure S2 Cell viability was analyzed after 24 h exposure to different concentrations of p, p'-DDE (A) and β-HCH (B). The results were expressed as a ratio relative to controls and presented as mean ± SEM of six independent experiments.

Supplement Table 1. Sequences of primers for RT-PCR.

Gene	Sequences
Mouse-Acc F	GCCGTGGGGAAGGAAAAGT
Mouse-Acc R	GTGGCTAGGTACTGAACAAAGAG
Mouse-Fas F	TATCAAGGAGGCCCATTTTGC
Mouse-Fas R	TGTTTCCACTTCTAAACCATGCT
Mouse-Scd1 F	TTCTTGCGATACACTCTGGTGC
Mouse-Scd1 R	CGGGATTGAATGTTCTTGTCGT
Mouse-Cpt1α F	ACGTTGGACGAATCGGAACA
Mouse-Cpt1α R	GGTGGCCATGACATACTCCC
Mouse-Acox1 F	CCGTCGAGAAATCGAGAACT
Mouse-Acox1 R	ATTGAGGCCAACAGGTTCCA
Mouse-Scad F	ATGTGCCAGAGGAGCTGAGT
Mouse-Scad R	TGATCCACTGTTGCTTCTGC
Mouse-Mcad F	AACTAAACATGGGCCAGCGA
Mouse-Mcad R	CAGCTGCGACTGTAGGTCTG
Mouse-Lcad F	GCATCAACATCGCAGAGAAA
Mouse-Lcad R	ACGCTTGCTCTTCCCAAGTA
Mouse-GAPDH F	AGGTCGGTGTGAACGGATTTG
Mouse-GAPDH R	TGTAGACCATGTAGTTGAGGTCA
Human-CPT1α F	ATCAATCGGACTCTGGAAACGG
Human-CPT1α R	TCAGGGAGTAGCGCATGGT
Human-SCAD F	CGGCAGTTACACACCATCTAC
Human-SCAD R	GCAATGGGAAACAACTCCTTCTC
Human-MCAD F	GGAAGCACATACCCCAGGAAT
Human-MCAD R	AGCTCCGTCACCAATTAAAACAT
Human-LCAD F	TGCAATAGCAATGACAGAGCC
Human-LCAD R	CGCAACTACAATCACAACATCAC
Human-GAPDH F	TGACAACTTTGGTATCGTGGAAGG
Human-GAPDH R	AGGCAGGGATGATGTTCTGGAGAG

F: forward; R: reverse

Supplement Table 2. Effect of p,p'-DDE and β-HCH on serum biochemical

Index	Control	p,p'-DDE	β-HCH
ALT (U/L)	38.6±10.5	52.9±10.7	28.8±5.8
AST (U/L)	78.9±3.9	80.2±27.7	53.4±8.6
TP (g/L)	56.9±1.2	54.1±1.1	56.6±0.7
ALB (g/L)	38.3±0.7	36.5±0.8	36.4±0.7
TBIL (umol/L)	1.9±0.3	1.1±0.3	1.1±1.1
ALP (U/L)	90.0±6.1	71.0±5.7	76.5±6.1
GLU (mmol/L)	11.0±0.9	9.6±0.5	12.1±0.5
BUN (mmol/L)	9.9±0.3	10.3±0.5	9.1±0.6
CREA (umol/L)	9.0±2.1	13.5±0.5	10.0±1.4
CHOL (mmol/L)	3.1±0.2	2.9±0.2	2.9±0.1
TG (mmol/L)	0.8±0.03	0.8±0.02	0.7±0.1
HDL-C (mmol/L)	2.5±0.1	2.3±0.2	2.3±0.1
LDL-C (mmol/L)	0.1±0.02	0.1±0.02	0.1±0.02
GLOB (g/L)	18.7±0.6	17.6±0.6	20.2±0.3*

parameters (mean ± SEM, n = 8/group).

Significance indicated by: *p < 0.05 compared with the control group.

Supplement Table 3. Metabolomics analysis of liver tissues from mouse exposed to OCPs

Metabolites	RT	mass	Metabolic pathway	Fold changes (DDE/Ctrl)	Fold changes (DDE/Ctrl)
Cinnamic acid	2.39	148.05	Amino acids metabolism	0.14↓	1.33↑
Xanthurenic acid	1.11	205.04	Tryptophan metabolism	0.37↓	4.52↑
Sphinganine	9.91	301.30	Sphingolipid metabolites	1.71↑	1.85↑
Phenylpyruvic acid	1.29	164.05	Phenylalanine metabolism	2.85↑	4.21↑
PG(P-16:0/14:1(9Z))	13.82	676.48	Phospholipids metabolism	0.52↓	1.34↑
PE(18:1(9Z)/0:0)	10.93	479.30	Phospholipids metabolism	2.17↑	2.00↑
PE(18:0/0:0)	10.23	481.32	Phospholipids metabolism	0.89↓	3.11↑
PC(O-18:0/0:0)	12.57	509.39	Phospholipids metabolism	0.51↓	0.46↓
PC(8:0/7:0)[	9.69	495.30	Phospholipids metabolism	1.65↑	1.94↑
PC(14:0/0:0)	9.67	467.30	Phospholipids metabolism	0.88↓	5.16↑
Palmitic amide	9.33	255.26	Fatty acid metabolism	2.59↑	2.58↑
PA(22:1(11Z)/0:0)	9.69	492.33	Phospholipids metabolism	1.58↑	1.94↑
PA(20:1(11Z)/0:0)	9.69	464.30	Phospholipids metabolism	1.62↑	2.37↑
Homocysteine	1.41	135.03	Amino acids metabolism	3.20↑	4.38↑
Histidine	0.62	155.07	Amino acids metabolism	0.79↓	2.03↑
Uric acid	62.72	168.03	Purine metabolism	0.41↓	0.56↓
PI(18:1(9Z)/0:0)	627.45	598.31	Phospholipids metabolism	2.44↑	1.86↑
Phenylalanine	142.54	165.08	Amino acids metabolism	4.16↑	3.38↑
Glucose 6-phosphate	38.97	260.03	Glucose metabolism	3.34↑	2.56↑
Xanthurenic acid	66.71	205.04	Tryptophan metabolism	2.51↑	14.14↑
Xanthine	66.08	152.03	Purine metabolism	0.84↓	14.46↑
PS(19:0/0:0)	589.58	539.32	Phospholipids metabolism	1.87↑	2.81↑
PG(18:1(9Z)/0:0)	672.96	510.30	Phospholipids metabolism	1.96↑	6.09↑
Glucose	56.92	180.06	Glucose metabolism	4.96↑	5.22↑
PI(16:0/0:0)	619.92	572.30	Phospholipids metabolism	4.58↑	5.78↑

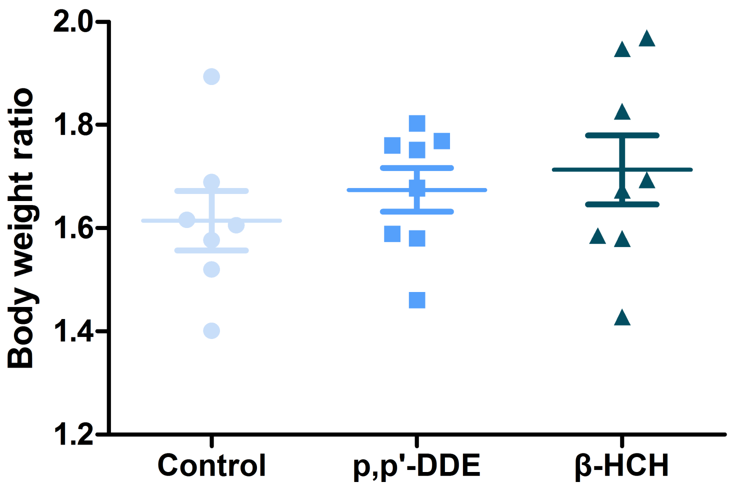

Figure S1

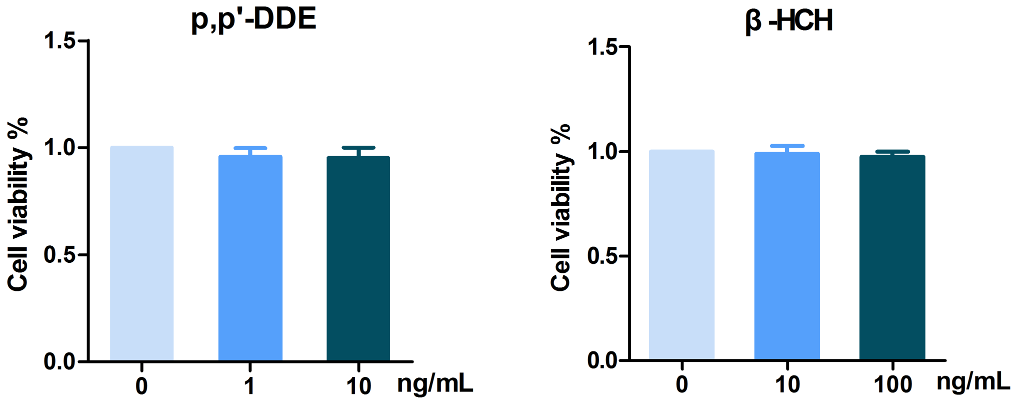

Figure S2
